# Malta Fever

**Published:** 1907-05-18

**Authors:** 


### Tropical Diseases.

### "MALTA FEVER."

Malta, Mediterranean, or undulant fever is a disease little seen by the ordinary practitioner in England, but though this is so it is an illness which has taken heavy toll of the naval and military men stationed from time to time in the Mediterranean, and has therefore cost the Government thousands of pounds in dealing with its effects. The first definite mention of the disease was made by medical officers of the two services in the early part of last century, but it was then more or less confused with remittent malaria, enteric fever, and other diseases, and nothing certain was known about its cause. In 1887, however, Colonel Bruce discovered the specific germ, a small micrococcus, and this discovery, coupled with his and Hughes' accurate accounts of the condition clinically, at once placed the literature of the disease on a firm basis. Later, when Widal's reaction for enteric fever became known, it was found that Malta fever also gave its own specific reaction; and this fact therefore rendered it easy to further differentiate those two conditions from each other. From 1887 onwards little more was done in trying to determine how this coccus gained entrance into man, or where it lived in nature, and the prevalence of the disease as far as Malta was concerned went on unabated. So severe were its ravages amongst the Services that at last the Government at home took definite action, and a Commission, appointed by the Admiralty, the War Office, and the Civil Government of Malta, under the supervision of an Advisory Committee of the Royal Society, was sent out to Malta to thoroughly investigate the disease. This Commission had the good fortune to have the services of Colonel Bruce, the Chairman of the Sub-Committee of the Royal Society, at their disposal, and work was definitely commenced in June 1904. For a considerable time ordinary routine work went on, a large mass of important material being collected, when suddenly a chance observation supplied the key to the mystery, and solved the riddle of how man becomes infected with the micrococcus melitensis. Some goats were being tested preparatory to being used for inoculation purposes one day, and it was then found that their blood-serum strongly clumped the specific germ—an observation which at once indicated that those animals were already infected with Malta fever; and so it proved, and not only so, but also that they excreted the cocci in enormous numbers in their milk. Feeding experiments on monkeys and other work soon proved the truth of this contention; and on the adoption of prophylactic measures an immediate fall in the number of cases at once became apparent. The most striking instance of this is related by Staff-Surgeon Clayton, one of the investigators. The Naval Hospital at Malta has always had a very bad reputation, fully one-third of the cases of Mediterranean fever among the seamen of the fleet at Malta being directly traceable to a residence in that institution. As soon as the discovery of the part the goat plays in the spread of the disease became known, the animals supplying the hospital were at once examined, and 10 per cent. of them were found to have the germs of the disease in their milk. The supply was at once stopped absolutely, and since that date not a single case of Malta fever has been traced to a residence there. This to anyone knowing the Naval Hospital at Malta is an extraordinary fact, and equally good results have been obtained in the military barracks throughout the island. Theoretically, therefore, if one could stamp out all the infected goats in Malta the disease would come to an end as far as that island is concerned; but practically there will be a considerable difficulty in doing this, chiefly owing to the opposition of the uneducated and often violently prejudiced natives of the agricultural class. It is computed that there are 20,000 goats in Malta supplying milk for the native and foreign community, and of those 2,000 or more are said to be infected. Two courses have been suggested by the Commissioners: to attempt, firstly, palliation of the disease; or, secondly, its complete eradication. The latter would seem to be the better of the two, but, as just stated, it will be difficult, and require very delicate handling by the civil authorities. Still, it would be a very great misfortune if such a brilliant discovery were to be made no use of, and it is to be sincerely hoped that the Home Government, the Admiralty, and the War Office will combine with the Civil Government of Malta in an attempt to utilise the facts, obtained after years of toil, for the amelioration, if nothing else, of the enormous amount of sickness that has so far prevailed amongst our men in Malta.

